# Frequency Domain Panoramic Imaging Algorithm for Ground-Based ArcSAR

**DOI:** 10.3390/s20247027

**Published:** 2020-12-08

**Authors:** Yun Lin, Yutong Liu, Yanping Wang, Shengbo Ye, Yuan Zhang, Yang Li, Wei Li, Hongquan Qu, Wen Hong

**Affiliations:** 1 School of Information Science and Technology, North China University of Technology, Beijing 100144, China; ylin@ncut.edu.cn (Y.L.); LiuYutong_ncut@outlook.com (Y.L.); zhangyuan@ncut.edu.cn (Y.Z.); liyang_ncut@ncut.edu.cn (Y.L.); lwsar@ncut.edu.cn (W.L.); qhqphd@ncut.edu.cn (H.Q.); 2 Aerospace Information Research Institute, Chinese Academy of Sciences, Beijing 100049, China; sbye@mail.ie.ac.cn (S.Y.); whong@mail.ie.ac.cn (W.H.)

**Keywords:** ground-based synthetic aperture radar (GBSAR), arc-scanning synthetic aperture radar (ArcSAR), SAR imaging algorithm, frequency domain algorithm, panoramic imaging

## Abstract

The ground-based arc-scanning synthetic aperture radar (ArcSAR) is capable of 360° scanning of the surroundings with the antenna fixed on a rotating arm. ArcSAR has much wider field of view when compared with conventional ground-based synthetic aperture radar (GBSAR) scanning on a linear rail. It has already been used in deformation monitoring applications. This paper mainly focuses on the accurate and fast imaging algorithms for ArcSAR. The curvature track makes the image focusing challenging and, in the classical frequency domain, fast imaging algorithms that are designed for linear rail SAR cannot be readily applied. This paper proposed an efficient frequency domain imaging algorithm for ArcSAR. The proposed algorithm takes advantage of the angular shift-invariant property of the ArcSAR signal, and it deduces the accurate matched filter in the angular-frequency domain, so panoramic images in polar coordinates with wide swath can be obtained at one time without segmenting strategy. When compared with existing ArcSAR frequency domain algorithms, the proposed algorithm is more accurate and efficient, because it has neither far range nor narrow beam antenna restrictions. The proposed method is validated by both simulation and real data. The results show that our algorithm brings the quality of image close to the time domain back-projection (BP) algorithm at a processing efficiency about two orders of magnitude better, and it has better image quality than the existing frequency domain Lee’s algorithm at a comparable processing speed.

## 1. Introduction

Synthetic Aperture Radar (SAR) is capable of high-resolution imaging in all-day and all-weather conditions. Besides, SAR images contain phase information, which can be used to extract deformation information by differential interferometry. It is a kind of non-contact and sub-wavelength high-precision deformation extraction technique. Ground-based synthetic aperture radar (GBSAR) is a type of SAR system that can continuously monitor the deformation of an area for a long period of time in real time [1,2,3,4,5]. It has been widely used in the deformation monitoring of dams, bridges, buildings, and slopes. This kind of technique, which is based on sensor scanning and imaging, is also widely employed for different applications, such as medical imaging and indoor mapping [6,7].

In a conventional GBSAR system, the antenna scans along a linear rail. Thus, the field of view is limited by the antenna beam width, and it cannot cover the whole surrounding areas for one observation. A new model of GBSAR, called arc-scanning synthetic aperture radar (ArcSAR), can overcome the above shortcoming. It is capable of 360° scanning of the surroundings with the antenna that is fixed on a rotating arm [8,9]. The same as linear rail SAR, its azimuth resolution is also determined by equivalent synthetic aperture. The length of the equivalent synthetic aperture is proportional to the beam width and the rotating radius. While, what is different is that the azimuth resolution does not degrade with squint angle, as in linear rail SAR. Currently, ArcSAR commercial products have already been developed [10], and they have been applied to the field of deformation monitoring.

In this paper, we mainly focus on the imaging algorithm for ArcSAR. The curvature track makes the image focusing challenging. Frequency domain fast algorithms that are designed for conventional linear rail GBSAR [4,5,11,12,13,14,15] cannot be readily applied to ArcSAR. Circular SAR also moves along a circular track, but it is often used in airborne platform, and the beam is spotted inwards from the circular track [16,17]. However, ArcSAR scans the surroundings outwards. The existing circular SAR algorithms cannot be readily applied to ArcSAR due to the difference in geometry. Although the time domain imaging algorithms, like the BP algorithm, are applicable for ArcSAR and can obtain high-precision imaging results [8], the heavy computation burden makes the algorithm time consuming, especially for high-resolution, wide-swath, 360° panoramic imaging.

There are already some frequency domain fast imaging algorithms for ArcSAR, but the approximation-made restricts their use to either narrow field of view or far range and narrow beam width conditions. The segmenting strategy is usually performed to realize wide-swath 360° panoramic imaging, which increases the computation complexity, and may have the problem of boundary discontinuity [18,19,20,21,22].

Luo et al. proposed a fast imaging algorithm [18,19]. They select a reference point in the scene, and then formulate its range cell migration (RCM) correction function and azimuth matched filter function. With their algorithm, the reference point can be well focused, while targets that are located far away from the reference point are defocused because of the signal spatial variant property. Accordingly, the effective imaging field of view of this algorithm is narrow, and the segmenting strategy is needed in order to obtain the 360°-scanning image.

Lee et al. proposed a RD algorithm for ArcSAR [20,21]. They formulate the time domain matched filter function for a reference range rather than a reference point, so that targets from the reference range but different aspect angles can all be well focused. However, in their formulation, the range migration is expanded by Taylor, and the terms higher than quadratic are ignored. This approximation can only be neglected under narrow beam width condition, while for wider beam width, defocusing will occur. Moreover, the range variant correction is not considered in this algorithm. Thus, targets from near range and far range may suffer from defocusing, which means that the well-focused swath is narrow. For wide-swath imaging, segmenting in range is needed.

In this paper, we propose a novel fast and accurate frequency domain imaging algorithm for ArcSAR panoramic imaging. Because it is performed in the frequency domain, the algorithm is of high computational efficiency. In our algorithm, the matched filter is accurately formulated, and the range-variant problem is also handled, so the algorithm is very accurate. A wide-swath 360° panoramic image can be obtained without any segmenting strategy. When compared with Luo’s algorithm, our algorithm can focus the 360° raw data at one time, and does not need to divide the data into narrow angle data. When compared with Lee’s RD algorithm, our algorithm does not use Taylor’s expansion, and it accurately formulates the frequency domain matched filter function for the referenced range. Thus, targets at reference range can be accurately focused without far range and narrow beam assumption. Moreover, the range variant correction function in the range and angular frequency domain is also accurately deduced. Therefore, not only the reference range targets, but also the near and far range targets, are all well focused, which means that the well-focused swath is wide.

The advantages of the proposed algorithm over the other algorithms are validated by both simulation and real data. The 360° field of view imaging capability is validated by simulation, but it is partially validated by the real data, because of the current ±80° scanning limitation of the system. The results, both simulation and real data, show that our algorithm brings the quality of image close to the time domain BP algorithm at a processing efficiency of approximately two orders of magnitude better, and it has better image quality than the existing frequency domain Lee’s algorithm at comparable processing speed.

This paper is organized, as follows. First, the geometry, the signal model, and the resolution of ArcSAR are introduced in Section 2. In Section 3, the proposed imaging algorithm is developed, and the errors that are caused by the algorithm are also analyzed. In Section 4, the proposed method is validated with both simulated data and real ArcSAR data, followed by concluding remarks in Section 5.

## 2. Geometry, Signal Model, and Resolution

### 2.1. Geometry of ArcSAR

**Figure 1:**
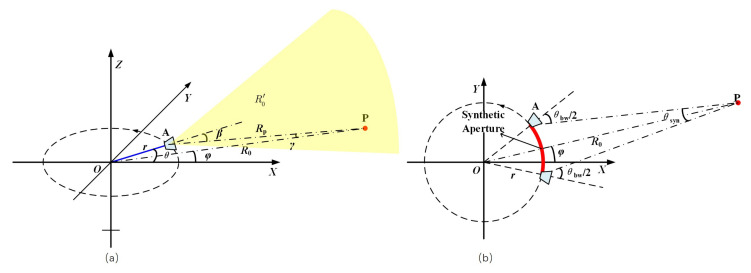
Geometry of arc-scanning synthetic aperture radar (ArcSAR): (**a**) Geometry of ArcSAR. (**b**) Top view of the ArcSAR geometry.

Figure 1 shows the geometry of ArcSAR. Antenna A is fixed on a rotating arm, and the beam of the antenna scans outwards in 360°. The radar system transmits and receives data at a constant pulse reputation time (PRT) as the antenna rotates. As shown in Figure 1a, the Cartesian coordinate system OXY is defined as the following: the antenna rotating center is defined as the origin O, and the rotating plane is defined as the OXY plane, and the Z axis points up.

In Figure 1a, *r* denotes the rotating radius and θ denotes the rotating angle. For the convenience of signal formulation, we assume that there is an point target P in the scene, which is located at the same plane of the rotating plane. The position of target P is expressed here in polar coordinates, as $\left(R_{0},\varphi \right)$, where $R_{0}$ denotes its distance to the rotating center and φ denotes its aspect angle. β denotes the instantaneous squint angle, which is the angle between the beam direction and line of sight. γ denotes the instantaneous angle between line PA and line PO. $R_{p}$ denotes the instantaneous range from target P to antenna phase center.

Figure 1b shows the top view of the ArcSAR geometry. The red arc in the figure represents the synthetic aperture of target P. When the antenna reaches the two ends of the synthetic aperture, the instantaneous squint angle β reaches $\pm \theta_{\mathrm{bw}}/2$, angle γ reaches $\pm \theta_{\mathrm{syn}}/2$, and the rotating angle θ reaches $\varphi \pm (\theta_{\mathrm{bw}}-\theta_{\mathrm{syn}})/2$, where $\theta_{\mathrm{bw}}$ denotes the beam width and $\theta_{\mathrm{syn}}$ denotes the synthetic aperture angle.

### 2.2. Signal Model of ArcSAR

The transmitted signal can be Frequency-Modulated Continuous Wave (FMCW), Chirp, stepped frequency signal, and so on. No matter what the waveform is, after preprocessing, the range compressed signal of echo from target P in the frequency domain can all be expressed as:

(1) $$s\left(\theta ,f\right)=\delta_{p}\cdot \mathrm{rect}\left(\frac{\theta -\varphi }{\theta_{\mathrm{bw}}-\theta_{\mathrm{syn}}}\right)\cdot \mathrm{rect}\left(\frac{f}{B_{r}}\right)\cdot \exp\left(-j\frac{4\pi (f+f_{c})}{C}R_{p}\right)$$

where *f*, $f_{c}$, and $B_{r}$ denote the baseband frequency, center frequency, and bandwidth of the transmitted signal, respectively. *C* denotes the speed of light and $\delta_{p}$ denotes the reflectivity of target P. $R_{p}$ denotes the instantaneous range from target P to the antenna phase center and, based on the triangle cosine theory, its expression is,

(2) $$R_{p}=\sqrt{R_{0}^{2}+r^{2}-2R_{0}r\cos\left(\theta -\varphi \right)}$$

Different from conventional SAR with a linear track, the range migration of ArcSAR is not a hyperbola, but it is approximately partial of a cosine curve.

For simplicity, we denote $K=4\pi (f+f_{c})/C$ as the two-way wavenumber and $K_{c}=4\pi f_{c}/C$ as the two-way center wavenumber. Rewrite (1) as a function of wavenumber as,

(3) $$s\left(\theta ,K\right)=\delta_{p}\cdot \mathrm{rect}\left(\frac{\theta -\varphi }{\theta_{\mathrm{bw}}-\theta_{\mathrm{syn}}}\right)\cdot \mathrm{rect}\left(\frac{K-K_{c}}{4\pi B_{r}/C}\right)\cdot \exp\left(-jK\cdot R_{p}\right)$$

### 2.3. Resolution of ArcSAR

The range resolution of ArcSAR is the same as linear rail GBSAR, and it is determined by the transmitted signal bandwidth, which is,

(4) $$\Delta_{r}=C/\left(2B_{r}\right)$$

The azimuth resolution is determined by the synthetic aperture angle $\theta_{\mathrm{syn}}$, as shown in Figure 1b. The azimuth resolution is,

(5) $$\Delta_{a}=\frac{\lambda_{c}}{4\sin(\theta_{\mathrm{syn}}/2)}$$

where $\lambda_{c}=C/f_{c}$ denotes the center wavelength.

In triangle OAP, as shown in Figure 1b, based on the triangle sine theory, we can easily have the following expression,

(6) $$\sin\left(\theta_{\mathrm{syn}}/2\right)=\frac{r\sin(\theta_{\mathrm{bw}}/2)}{R_{0}}$$

From (6), we can see that the synthetic aperture angle of ArcSAR is proportional to the rotating radius *r* and the beam width $\theta_{\mathrm{bw}}$.

Substitute (6) into (5), we have the azimuth resolution equation, as

(7) $$\Delta_{a}=\frac{\lambda_{c}R_{0}}{4r\sin(\theta_{\mathrm{bw}}/2)}$$

Thus, the angular resolution can be easily formulated as,

(8) $$\Delta_{\theta }=\Delta_{a}/R_{0}=\frac{\lambda_{c}}{4r\sin(\theta_{\mathrm{bw}}/2)}$$

From (7), we can see that the azimuth resolution gets worse with increasing range and, from (8), we can see that the angular resolution is constant with range.

## 3. Frequency Domain Panoramic Imaging Algorithm

The signal of ArcSAR has angular shift invariant and range variant property. From (2), its easy to know that targets from the same range $R_{0}$, but different aspect angle φ have the same range migration curves that are shifted in aspect angle only, and this is called angular shift invariant property. Additionally, as indicated in (2), the range migration curves are different for targets at different range $R_{0}$, which is called range variant property.

In this paper, we take advantage of the angular shift invariant property of the ArcSAR signal, and the main steps take place in the angular frequency domain. In the angular frequency domain, signals of targets at the same range, but different aspect angles, overlap, which makes fast imaging possible.

The main two steps of our algorithm are two-dimensional (2-D) matched filtering in the 2-D frequency domain, and range variant compensation in the range and angular frequency domain. The 2-D matched filter is accurately formulated for a chosen reference range, so that the signals of targets from the reference range, but different aspect angles, can all be accurately matched. The differential phase correction function is deduced in the range and angular frequency domain, so that the correction can change with range in order to deal with the range-variant problem.

The output image is in polar coordinates because our algorithm is performed in the angular frequency domain, and the angular frequency corresponds to the rotating angle in space domain.

### 3.1. 2-D Frequency Domain Signal

The 2-D frequency domain signal is first deduced in this paper because it helps to design the matched filter and range variant correction function. In this session, we will deduce the accurate explicit expression of the 2-D frequency domain signal for ArcSAR for the first time.

As mentioned above, the ArcSAR signal has angular shift invariant property. For convenience, here, we set the aspect angle φ of point target P to zero, because aspect angle φ only contributes as a linear phase term $\exp\left(-jK_{\theta }\varphi \right)$ in the angular frequency domain, where $K_{\theta }$ is the angular wavenumber.

Substituting φ = 0 into (2) and (3), we can rewrite the instantaneous range $R_{p}$ and echo signal s(θ,K), as

(9) $$R_{p}=\sqrt{R_{0}^{2}+r^{2}-2R_{0}r\cos\theta }$$

and

(10) $$s\left(\theta ,K\right)=\delta_{p}\cdot \mathrm{rect}\left(\frac{\theta }{\theta_{\mathrm{bw}}-\theta_{\mathrm{syn}}}\right)\cdot \mathrm{rect}\left(\frac{K-K_{c}}{4\pi B_{r}/C}\right)\cdot \exp\left(-jK\cdot R_{p}\right)$$

respectively.

For the rest of the paper, the constant amplitude in formulation, like $\delta_{p}$, will be neglected for simplicity. While performing angular Fourier Transform to (10), we have the 2-D frequency domain signal, as,

(11) $$\begin{array}{c} S_{2\mathrm{df}}\left(K_{\theta },K\right)=\int_{-(\theta_{\mathrm{bw}}-\theta_{\mathrm{syn}})/2}^{(\theta_{\mathrm{bw}}-\theta_{\mathrm{syn}})/2}s\left(\theta ,K\right)\cdot \exp\left(-jK_{\theta }\theta \right)d\theta \\ =\int_{-(\theta_{\mathrm{bw}}-\theta_{\mathrm{syn}})/2}^{(\theta_{\mathrm{bw}}-\theta_{\mathrm{syn}})/2}\mathrm{rect}\left(\frac{K-K_{c}}{4\pi B_{r}/C}\right)\cdot \exp\left(j\psi (\theta )\right)d\theta \end{array}$$

where phase ψ has the expression of,

(12) $$\psi \left(\theta \right)=-KR_{p}\left(\theta \right)-K_{\theta }\theta$$

According to the principle of stationary phase (PSP), the stationary point satisfies dψ(θ)/dθ=0, so that the stationary point is,

(13) $$K_{\theta }=-K\cdot \frac{dR_{p}\left(\theta \right)}{d\theta }=-K\frac{rR_{0}\sin\theta }{R_{p}\left(\theta \right)}$$

Equation (13) provides the time-frequency relation between θ and $K_{\theta }$.

According to the PSP, the 2-D frequency signal has the form of,

(14) $$\begin{array}{c} S_{2\mathrm{df}}\left(K_{\theta },K\right)=\mathrm{rect}\left(\frac{K_{\theta }}{B_{\theta }}\right)\cdot \mathrm{rect}\left(\frac{K-K_{c}}{4\pi B_{r}/C}\right)\cdot \exp\left\{j\psi \left(\theta (K_{\theta })\right)\right\}\\ =\mathrm{rect}\left(\frac{K_{\theta }}{B_{\theta }}\right)\cdot \mathrm{rect}\left(\frac{K-K_{c}}{4\pi B_{r}/C}\right)\cdot \exp\left\{-jK\cdot R_{p}\left(\theta (K_{\theta })\right)-jK_{\theta }\cdot \theta \left(K_{\theta }\right)\right\} \end{array}$$

where $B_{\theta }$ denotes the angular bandwidth in wavenumber, and its expression is,

(15) $$B_{\theta }=2\pi /\Delta_{\theta }=8\pi r\sin\left(\theta_{\mathrm{bw}}/2\right)/\lambda_{c}$$

In (9), because $R_{p}$ is a complicated function of θ, we cannot obtain the explicit expression of θ with respect to $K_{\theta }$ easily just with (13), and we will deduce it in the next subsection.

For focusing, our aim is to turn (14) into the form of,

(16) $$S_{2\mathrm{dfc}}\left(K_{\theta },K\right)=\mathrm{rect}\left(\frac{K_{\theta }}{B_{\theta }}\right)\cdot \mathrm{rect}\left(\frac{K-K_{c}}{4\pi B_{r}/C}\right)\cdot \exp\left\{-jK\cdot R_{0}\right\}$$

with a matched filter, so that the phase term only contains a linear phase related to the position of the target, and the target will be focused in polar coordinates at position $\left(R_{0},\varphi \right)$ after 2-D Inverse Fourier Transform.

What we need to do next is formulate the 2-D matched filter in the 2-D frequency domain.

### 3.2. 2-D Matched Filter in the 2-D Frequency Domain

With (14) and (16), we know that the 2-D matched filter needs to have the form of,

(17) $$\begin{array}{c} H_{2df}\left(K_{\theta },K\right)=S_{2\mathrm{dfc}}\left(K_{\theta },K\right)\cdot S_{2df}^{*}\left(K_{\theta },K\right)\\ =\exp\left\{jK\cdot \left(R_{p}\left(\theta (K_{\theta })\right)-R_{0}\right)+jK_{\theta }\cdot \theta \left(K_{\theta }\right)\right\} \end{array}$$

where the superscript symbol * represents conjugate operation. In the 2-D frequency domain, the matched filter function can only contain two variables, $K_{\theta }$ and *K*. We need to substitute θ with the function of $K_{\theta }$.

As we know, for conventional SAR with a linear track, the signal in azimuth is approximately a linear frequency modulated signal, while, for ArcSAR, it is a different case. With the PSP, we have the angular wavenumber $K_{\theta }$ as a function of rotating angle θ, as shown in (13). However, it is hard to formulate an explicit expression of θ with respect to $K_{\theta }$ just with (13), because $R_{p}$ is also a function of θ. Accordingly, how to replace θ with function of $K_{\theta }$ in (17) is the key to the formulation of the matched filter for ArcSAR. Here, we use the triangle sine theory in order to solve this problem.

Based on the triangle sine theory, in triangle OAP, as shown in Figure 1a, we have,

(18) $$\frac{R_{p}}{\sin\theta }=\frac{R_{0}}{\sin\beta }=\frac{r}{\sin(\beta -\theta )}$$

and, then, we can easily have,

(19) $$\sin\beta =R_{0}\sin\theta /R_{p}$$

and

(20) $$\theta =\beta -\arcsin(r\sin\beta /R_{0})$$

respectively.

Substituting (19) into (13), we have $K_{\theta }$ as a function of squint angle β, as,

(21) $$K_{\theta }=-K\cdot r\cdot \sin\beta$$

Thus, β as a function of $K_{\theta }$ can be expressed as,

(22) $$\beta =-\arcsin\left(\frac{K_{\theta }}{K\cdot r}\right)$$

Equations (20) and (22) are important, because (20) provides the explicit expression of θ with respect to the instantaneous squint angle β, and (22) provides the explicit expression of β with respect to $K_{\theta }$.

Substituting (22) into (20), we have

(23) $$\theta \left(K_{\theta }\right)=-\arcsin\left\{\frac{K_{\theta }}{K\cdot r}\right\}+\arcsin\left\{\frac{K_{\theta }}{K\cdot R_{0}}\right\}$$

Now, the explicit expression of θ as a function of $K_{\theta }$ for ArcSAR is deduced. Substituting (23) into (14), the accurate expression of the 2-D frequency domain signal can be obtained.

Substituting (23) into (17), the accurate matched filter for targets at range $R_{0}$ is deduced without any approximation.

Because the target range $R_{0}$ is unknown, we replace $R_{0}$ with the reference range $R_{c}$ in the matched filter, so that targets at the reference range can all be accurately focused. The expression of the 2-D matched filter in the 2-D frequency domain is,

(24) $$H_{2df}\left(K_{\theta },K\right)=\exp\left\{jK\cdot \left(R_{p}\left(K_{\theta },R_{0}=R_{c}\right)-R_{c}\right)\right.\left.+jK_{\theta }\cdot \theta \left(K_{\theta },R_{0}=R_{c}\right)\right\}$$

where,

(25) $$R_{p}\left(K_{\theta },R_{0}=R_{c}\right)=\sqrt{R_{c}^{2}+r^{2}-2R_{c}r\cos\left(\theta (K_{\theta },R_{0}=R_{c})\right)}$$

and

(26) $$\theta \left(K_{\theta },R_{0}=R_{c}\right)=-\arcsin\left\{\frac{K_{\theta }}{K\cdot r}\right\}+\arcsin\left\{\frac{K_{\theta }}{K\cdot R_{c}}\right\}$$

### 3.3. Range Variant Correction Function in the Range and Angular Frequency Domain

In the formulation of the matched filter previously, because the target range $R_{0}$ is unknown in the 2-D frequency domain, it is replaced by reference range $R_{c}$. Thus, targets at reference range $R_{c}$ can be accurately focused, while targets at other ranges still have a differential phase to be corrected.

In this section, the range variant correction function will be deduced in the range and angular frequency domain, because, in this domain, the correction function can change with range.

When multiplying the 2-D frequency domain signal (14) with the 2-D matched filter (24), the signal turns to,

(27) $$\begin{array}{c} S_{2\mathrm{dfc}}\left(K_{\theta },K\right)=S_{2\mathrm{df}}\left(K_{\theta },K\right)\cdot H_{2df}\left(K_{\theta },K\right)\\ =\mathrm{rect}\left(\frac{K_{\theta }}{B_{\theta }}\right)\cdot \mathrm{rect}\left(\frac{K-K_{c}}{4\pi B_{r}/C}\right)\cdot \exp\left\{-jKR_{0}+j\Phi_{\mathrm{dif}}\right\} \end{array}$$

where $\Phi_{\mathrm{dif}}$ denotes the differential phase that is caused by range variance. $\Phi_{\mathrm{dif}}$ is the phase difference between (17) and (24), and its expression is,

(28) $$\begin{array}{c} \Phi_{\mathrm{dif}}\left(K_{\theta },K\right)\\ =K\left(R_{p}\left(K_{\theta },R_{0}=R_{c}\right)-R_{c}\right)+K_{\theta }\cdot \theta \left(K_{\theta },R_{0}=R_{c}\right)-K\left(R_{p}\left(K_{\theta },R_{0}\right)-R_{0}\right)-K_{\theta }\cdot \theta \left(K_{\theta },R_{0}\right)\\ =K\cdot R_{\mathrm{dif}}+K_{\theta }\left(\theta \left(K_{\theta },R_{0}=R_{c}\right)-\theta \left(K_{\theta },R_{0}\right)\right) \end{array}$$

where $R_{\mathrm{dif}}$ presents the differential RCM, and it has the expression of,

(29) $$R_{\mathrm{dif}}=R_{p}\left(K_{\theta },R_{0}=R_{c}\right)-R_{c}-R_{p}\left(K_{\theta },R_{0}\right)+R_{0}$$

Here, we make an approximation, which is the only approximation made in our proposed algorithm. From (25) and (26), we can see that in the 2-D frequency domain, $R_{p}$ and θ, are not only a function of $K_{\theta }$, but also a function of *K*. To decouple *K* and $K_{\theta }$ in $R_{p}$ and θ, we replace variable *K* with center wavenumber $K_{c}$ for $R_{p}$ and θ, then (28) turns to,

(30) $$\begin{array}{c} \Phi_{\mathrm{dif}}\left(K_{\theta },K\right)\\ \approx jK\cdot R_{\mathrm{dif}}\left(K=K_{c}\right)+jK_{\theta }\left(\theta \left(K_{\theta },R_{0}=R_{c},K=K_{c}\right)-\theta \left(K_{\theta },R_{0},K=K_{c}\right)\right)\\ =j\left(K-K_{c}\right)\cdot R_{\mathrm{dif}}\left(K=K_{c}\right)+\Phi_{\mathrm{dif}}\left(K_{\theta },K=K_{c}\right) \end{array}$$

The first term of the right side of (30) is the linear phase term for the differential RCM, and the second term is the differential phase term.

Apply the range Inverse Fourier Transform to (27), then we have the range and angular frequency domain signal, as

(31) $$S_{\mathrm{rd}}\left(K_{\theta },R\right)\approx \mathrm{rect}\left(\frac{K_{\theta }}{B_{\theta }}\right)\cdot \mathrm{sinc}\left\{\frac{2B_{r}}{C}\left(R-R_{0}+R_{\mathrm{dif}}\right)\right\}\cdot \exp\left\{-jK_{c}\cdot R_{0}+j\Phi_{\mathrm{dif}}\left(K_{\theta },K=K_{c}\right)\right\}$$

If the differential RCM exceeds half a range cell, then it better not be ignored, and it should be corrected in the range and angular frequency domain by range re-sampling, before the range-variant differential phase correction process.

The differential phase compensation function changes with range and its expression is,

(32) $$H_{\mathrm{dif}}\left(K_{\theta },R\right)=\exp\left\{-j\Phi_{\mathrm{dif}}\left(K_{\theta },K=K_{c},R_{0}=R\right)\right\}$$

From (32), we can see that the differential phase compensation function changes with range in the range and angular frequency domain, so that the range-variant differential phase for the whole swath can be accurately compensated.

### 3.4. Algorithm Flow

**Figure 2:**
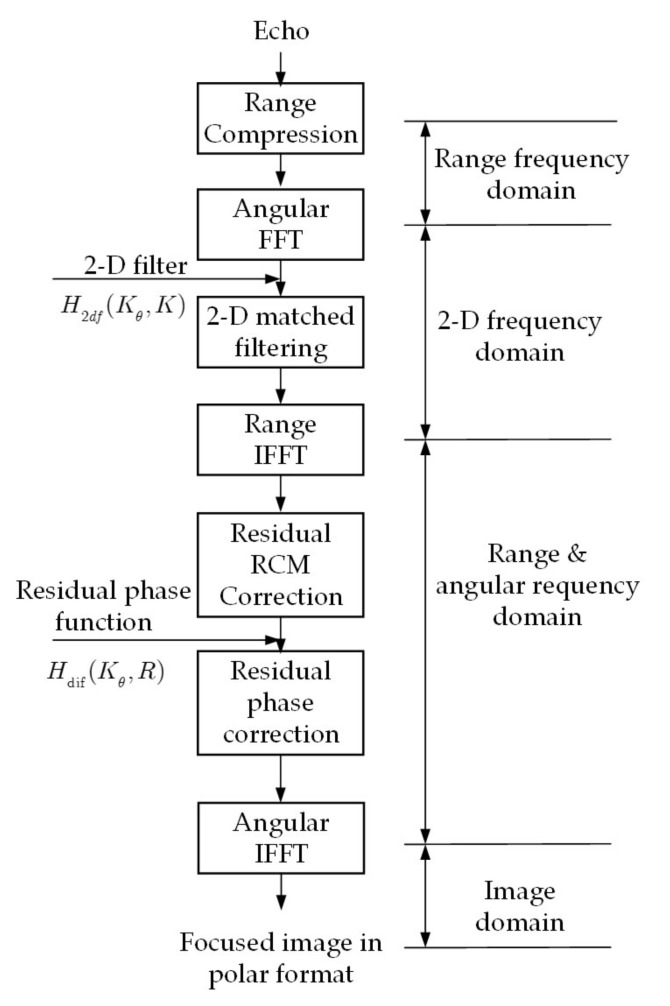
Flow chart of the proposed ArcSAR imaging algorithm.

Figure 2 shows the flow chart of the proposed method. The processing steps are described in details, as follows.

Step 1: Range compression. In this step, the signal is in angular and range-frequency domain.

Step 2: Angular Fast Fourier Transform (FFT). After angular FFT, the signal is in the 2-D frequency domain.

Step 3: Matched filtering. Multiply the 2-D frequency domain signal with the 2-D matched filter $H_{2df}\left(K_{\theta },K\right)$, which is accurate for reference range. The reference range is recommended to choose the range center.

Step 4: Range Inverse FFT (IFFT). After range IFFT, the signal is now transformed into the range and angular frequency domain.

Step 5: Differential RCM correction. Correct the range-variant differential RCM by range resampling. This step can be skipped for computing efficiency if the differential RCM is within half a range cell.

Step 6: Differential phase compensation. Multiply the signal with the differential phase compensation function $H_{\mathrm{dif}}\left(K_{\theta },R\right)$.

Step 7: Angular IFFT. After angular IFFT, the image is accurately focused in polar coordinates. If necessary, an interpolation algorithm could be used in order to convert the polar samples into rectilinear samples.

### 3.5. Errors Caused by the Proposed Algorithm

The only approximation made in the proposed algorithm is in (30). In (30), the variable *K* is replaced with center wavenumber $K_{c}$ for $R_{p}$ and θ, in order to decouple *K* and $K_{\theta }$, and prepare for the formulation of the differential phase correction function in the range and angular frequency domain. The phase error can be expressed as the following,

(33) $$\begin{array}{c} \Phi_{error}=K\left(R_{dif}\left(K\right)-R_{dif}\left(K=K_{c}\right)\right)\\ +K_{\theta }\left(\theta \left(K_{\theta },R_{0}=R_{c},K\right)-\theta \left(K_{\theta },R_{0},K\right)\right)\\ -K_{\theta }\left(\theta \left(K_{\theta },R_{0}=R_{c},K=K_{c}\right)-\theta \left(K_{\theta },R_{0},K=K_{c}\right)\right) \end{array}$$

From (25) and (26), we can see that, for $R_{p}$ and θ, the variable *K* is in denominator, so the phase error that is caused by our approximation is very small, which will be analyzed in the experiments section.

### 3.6. Errors Caused by Reference Plane Imaging

Unlike linear track SAR, which focuses the image in the slant plane without the need of terrain information, ArcSAR has a curvature track, and the terrain of the scene has an impact on image focusing.

In this algorithm, the terrain of the scene is not considered, and the rotating plane is selected as the reference plane for imaging. For targets that are located at the reference plane, the algorithm is accurate, while, for targets not located at the reference plane, phase error will occur. In this section, we will discuss the phase error that is caused by focusing on a fixed reference plane.

**Figure 3:**
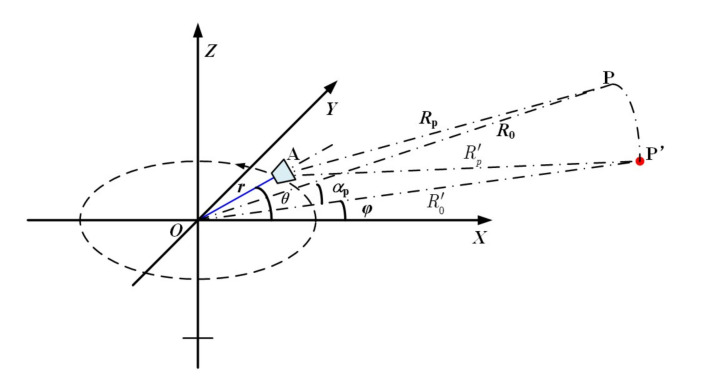
Target with height and its projection on the imaging plane.

As shown in Figure 3, target P is now not on the rotating plane, but with elevation angle $\alpha_{p}$. Its range to the rotating center is still denoted as $R_{0}$, thus its three-dimensional (3-D) coordinates are:

(34) $$\mathrm{Position}\left(P\right)=\left(R_{0}\cos\alpha_{p}\cos\varphi ,R_{0}\cos\alpha_{p}\sin\varphi ,R_{0}\sin\alpha_{p}\right)$$

Position P’ is on the rotating plane, and it has the same nearest range to the antenna phase center and the same aspect angle as target P. Thus, the coordinates of position P’ are:

(35) $$\mathrm{Position}\left(P^{\prime }\right)=\left(R_{0}^{\prime }\cos\varphi ,R_{0}^{\prime }\sin\varphi ,0\right)$$

where $R_{0}^{\prime }$ represents range from P’ to the rotating center, and its expression is,

(36) $$R_{0}^{\prime }=\sqrt{R_{0}^{2}+r^{2}-2rR_{0}\cos\alpha_{p}}+r\approx R_{0}+r\left(1-\cos\alpha_{p}\right)$$

According to the equal-range and equal-doppler principle in SAR imaging, target P will be projected to P’ in the output image if the rotating plane is chosen as the imaging plane. Position P and P’ havee the same nearest range to the antenna phase center, but with different height, thus the RCM difference between position P and position P’ will cause imaging error.

Still, for simplicity, the aspect angle φ of target P is assumed to be zero, then the instantaneous range from position P to the antenna phase center is,

(37) $$R_{p}=\sqrt{R_{0}^{2}+r^{2}-2R_{0}r\cos\theta \cos\alpha_{p}}\approx R_{0}-r\cos\theta \cos\alpha_{p}$$

While, the instantaneous range from position P’ to the antenna phase center is,

(38) $$R_{p}^{\prime }=\sqrt{{R_{0}^{\prime }}^{2}+r^{2}-2R_{0}^{\prime }r\cos\theta }\approx R_{0}^{\prime }-r\cos\theta \approx R_{0}+r\left(1-\cos\alpha_{p}\right)-r\cos\theta$$

Thus, the phase error that is caused by RCM error is,

(39) $$\Phi_{\mathrm{error}\_\mathrm{height}}=K\cdot \left(R_{p}-R_{p}^{\prime }\right)\approx -K\cdot r\cdot \left(1-\cos\theta \right)\cdot \left(1-\cos\alpha_{p}\right)$$

The maximum phase error tolerant for a high quality focusing should be kept within π/4 [23]. The maximum phase error occurs at the two ends of the synthetic aperture that are shown in Figure 1b, and the constraint is,

(40) $$\begin{array}{c} {\left|\Phi_{\mathrm{error}\_\mathrm{height}}\right|}_{\max}=\left|\Phi_{\mathrm{error}\_\mathrm{height}}\left(K=K_{\max},\theta =\pm \theta_{bw}/2\right)\right|\\ \approx K_{\max}\cdot r\cdot \left(1-\cos\left(\theta_{bw}/2\right)\right)\cdot \left(1-\cos\alpha_{p}\right)\leq \pi /4 \end{array}$$

Accordingly, approximately, the elevation angle $\alpha_{p}$ should satisfy the following constraint in our algorithm,

(41) $$\left|\alpha_{p}\right|<\arccos\left(1-\frac{\pi }{4K_{\max}\cdot r\cdot \left(1-\cos\left(\theta_{\mathrm{bw}}/2\right)\right)}\right)$$

## 4. Experiments

### 4.1. Simulation

**Figure 4:**
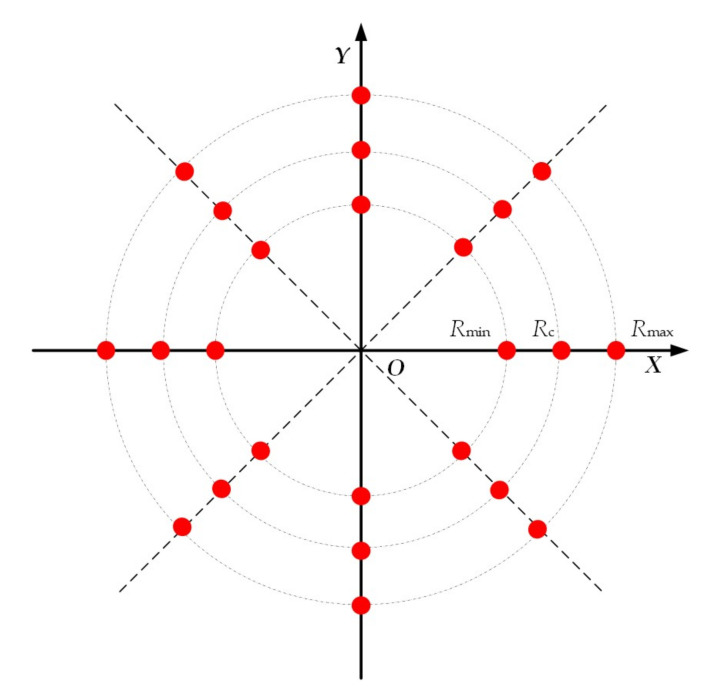
Distribution of the point targets for simulation.

Table 1 lists the simulated parameters. Figure 4 shows the distribution of the 24 point targets for simulation, where the near range $R_{\min}$, center range $R_{c}$, and far range $R_{\max}$ are 10 m, 500 m, and 1000 m, respectively.

In this simulation, we select the center range $R_{c}$ as the reference range.

**Figure 5:**
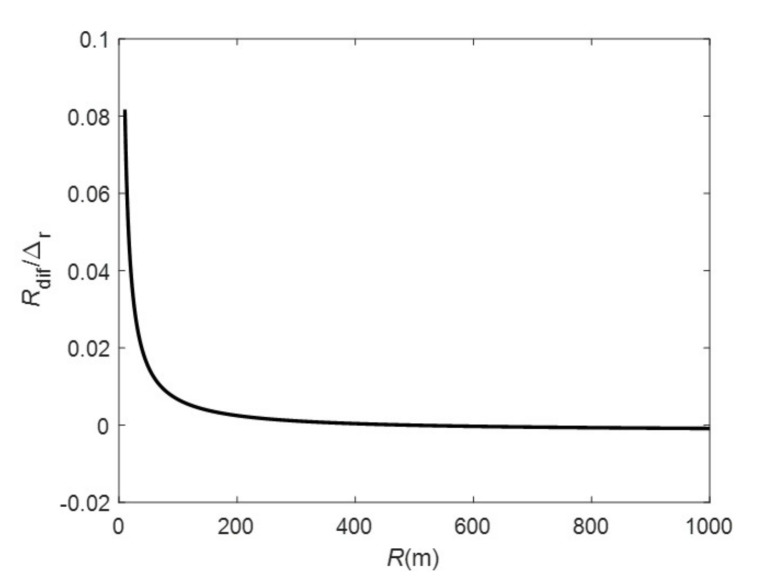
Differential RCM curve.

First, we analyze the errors of our algorithm with the given parameters. Figure 5 shows the curve of differential RCM $R_{\mathrm{dif}}$ with respect to range, according to (29). We can see that the differential RCM reaches its maximum at the near range, but it still does not exceeds half a range cell, so, in this simulation, the range re-sampling step in the range and angular frequency domain can be skipped for efficiency.

**Figure 6:**
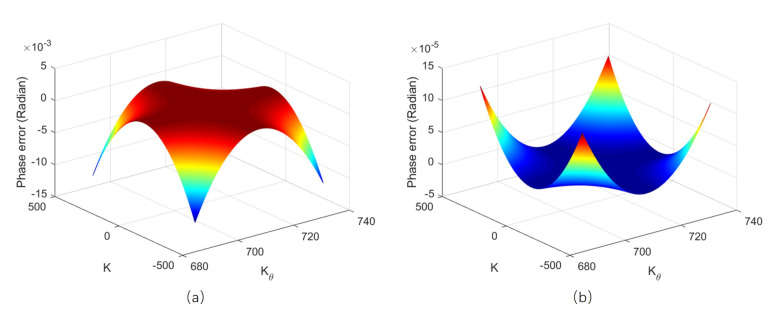
Phase error caused by the algorithm: (**a**) Phase error of targets at near range. (**b**) Phase error of targets at far range.

The phase error of target at center range is zero, because the matched filter is accurately formulated for the reference range. Here, the phase errors that are calculated with (33) of targets at near range and far range are shown in Figure 6. We can see that, even for the near target at 10 m, the phase error is still far smaller than π/4 and, for far range target, the phase error is even smaller. Therefore, our algorithm is very accurate for both near and far range imaging, and no segmenting strategy is needed.

To analyze the phase error that are caused by reference plane imaging, we substitute the simulation parameters into (41). For an optimal focus, the threshold that is calculated of the elevation angle is approximately 7.25°. That is to say, if the depression angle $\alpha_{p}$ exceeds the value, then a new imaging plane should be selected.

**Figure 7:**
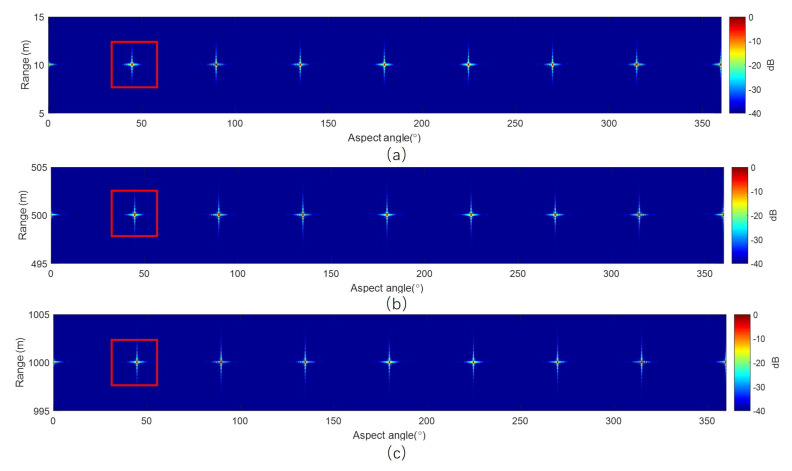
Imaging results with our algorithm: (**a**) Image of targets at near range. (**b**) Image of targets at center range. (**c**) Image of targets at far range.

**Figure 8:**
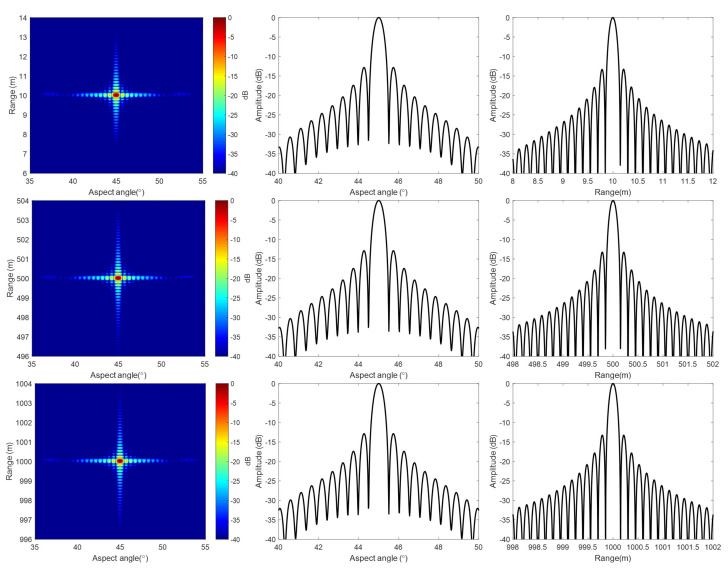
Details of the results with our algorithm: (**Up row**), near range target. (**Middle row**), center range target. (**Bottom row**), far range target. (**Left column**), expended 2-D image. (**Middle column**), azimuth profile through the pulse peak. (**Right column**), range profile through the pulse peak.

Figure 7 shows the imaging results of the simulation with our algorithm. The image is in polar coordinates, and the whole 360° scanning data are focused just for one imaging process, and no segmenting strategy is used. To clearly show the imaging results of near, center, and far range, targets are shown separately. Figure 8 shows the image details, as well as the range and aspect profiles. In polar coordinates, the point spread function is approximately a 2-D sinc function. The point spread function in polar coordinates for the whole swath is the same, because the angular resolution and the range resolution are constant with range.

**Figure 9:**
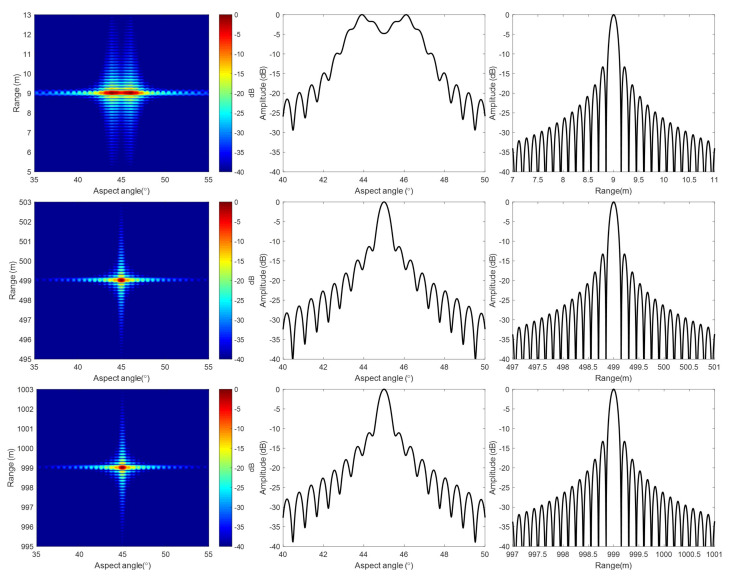
Details of the results with Lee’s algorithm: (**Up row**), near range target. (**Middle row**), center range target. (**Bottom row**), far range target. (**Left column**), expended two-dimensional (2-D) image. (**Middle column**), azimuth profile through the pulse peak. (**Right column**), range profile through the pulse peak.

For comparison, Figure 9 also shows the detailed target images and profiles of Lee’s algorithm. In Lee’s algorithm, the 2-D matched filter is in time domain, and it is formulated for a selected reference range. However, in the formulation, Taylor expansion with respect to the aspect angle is used, and the terms that are higher than quadratic are ignored. This approximation may cause defocusing, even for targets at the reference range under wide beam or near range condition. Defocusing is even more severe for targets not at the reference range.

**Figure 10:**
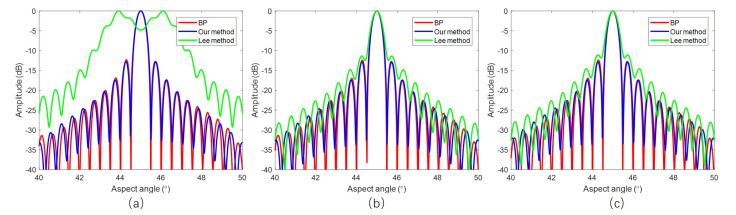
Azimuth profiles: (**a**) Profiles of Near range target. (**b**) Profiles of center range target. (**c**) Profiles of far range target.

We choose the BP algorithm as a benchmark. BP is based on pixel-by-pixel processing, and it is very accurate, although time consuming. Figure 10 compares the angular profiles of these three different algorithms. It shows that the image quality of our method is close to that of BP.

When comparing our results with that of Lee’s, we can see that, with our method, the whole swath is well focused. While, with Lee’s method, the near range targets are severely defocused, and the main lobe is split.

The point spread function of ArcSAR in polar coordinates is approximately a 2-D sinc function. We measure the 3 dB impulse response width (IRW), peak sidelobe ratio (PSLR), and integrated sidelobe ratio (ISLR) of the three targets marked with red rectangles in Figure 7 in order to test the imaging quality quantitatively. Because the algorithm induced defocusing occurs in angular direction, the quality test is only done in an angular direction for a concise purpose.

Table 2 shows the results of imaging quality test. With (8), the angular resolution is calculated as 0.5056°, and the 3 dB pulse width should be that value multiplied by 0.886, and that is 0.4479°. The tested angular resolution is about 0.4656° with our algorithm, and it is very close to the theoretical value. From (8), we also know that the azimuth resolution is the angular resolution multiplied by the target range. Thus, in our simulation example, the azimuth resolution of the near range, center range, and far range is 0.08 m, 4.06 m, and 8.12 m, respectively. The image quality is tested for BP result as a benchmark. The quality test results are also compared with that of Lee’s method. Need to mention that, for the near range target, the main lobe is split with Lee’s method, so the image quality is not measured. From the table, we can see that our method is very accurate for the whole swath.

The computing efficiency is also tested under the same environment. The codes of the three algorithms (in MATLAB language) have been implemented on the computer with 1.8-GHz i7-8550U CPU. The running times of our method, Lee’s method, and BP are 1.2414 s, 1.0108 s, and 693.5731 s, respectively.

### 4.2. Real Data

The ArcSAR system that is used in this field test was developed by our lab. It is still an experimental system, and the rotating angle is not 360, but about ±80°. This system uses frequency-modulated continuous wave (FMCW) and intermediate frequency (IF) receiver. Table 3 lists the main parameters of the system, where $f_{s}$ denotes the analogue to digital (AD) sampling rate and $T_{p}$ denotes the pulse duration. The unambiguous range is equal to $f_{s}T_{p}C/\left(4B_{r}\right)$, and that is about 900 m.

**Figure 11:**
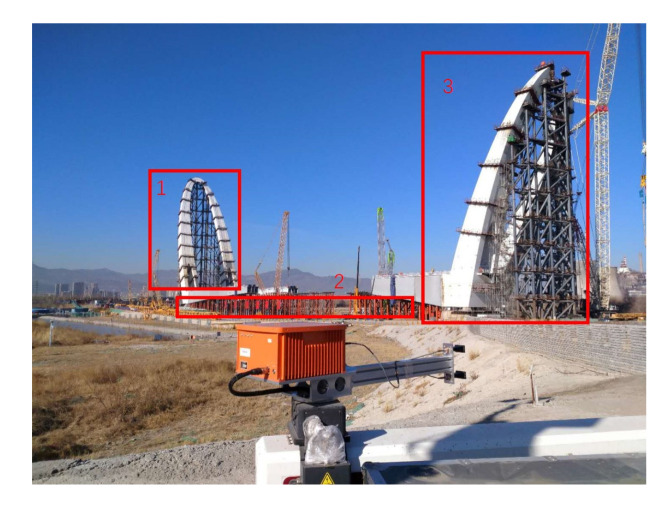
Photo of the ArcSAR system and the observed scene.

In this experiment, the observed scene is a bridge under construction. Figure 11 shows the photo of the experimental ArcSAR system and the observed scene. During data acquisition, only the arm rotates in order to scan the scene.

**Figure 12:**
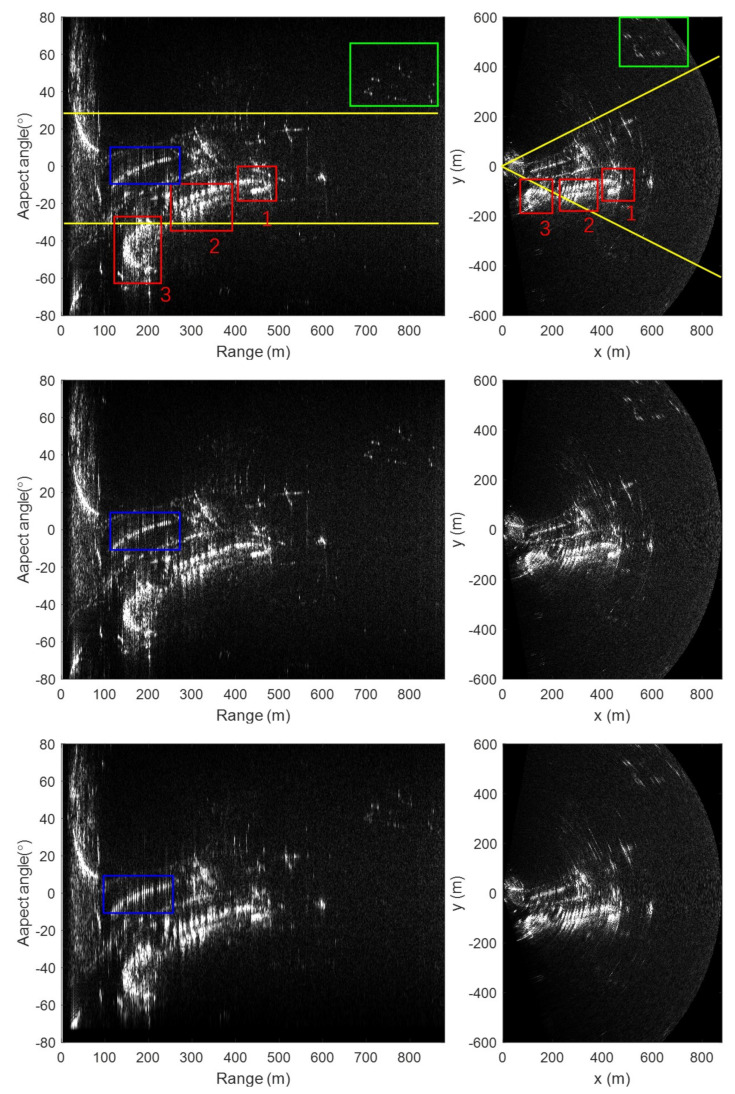
Real data imaging results: (**Up row**), Results with BP. (**Middle row**), Results with our method. (**Bottom row**), Results with Lee’s method. (**Left column**), Image in polar coordinates. (**Right column**), Image in Cartesian coordinates.

With our algorithm, the echo data can be focused accurately and fast. The left column of Figure 12 shows the output images of three algorithms in polar coordinates. We can see the 2-D sinc spread function of strong point-like targets in the image with our algorithm, indicating an optimal focus. For a better display, the polar coordinates image is transformed into the Cartesian coordinates, as shown in the right column. The deterioration of the image in Cartesian coordinates is not due to the focusing method, but rather to the limitation of $\theta_{syn}$ introduced earlier in Figure 1b. The correspondence between the ArcSAR image and the photo are labelled with the same number. Label 1 and label 3 represent the two towers of the bridge, respectively, and label 2 represents the slogan made of iron. The region between the two yellow lines in the figure indicates ±30° field of view limit that is covered by linear-scan GBSAR with 60° beamwidth antenna.

We can see that the results of our method are close to that of BP. Both near-range target (in the blue rectangle) and far-range target (in the green rectangle) are well focused. The image is defocused with Lee’s method, especially for the near-range.

In the same environment mentioned in the simulation section, the running times of our method, Lee’s method, and BP are 0.2448 s, 0.1833 s, and 46.1571 s, respectively.

## 5. Conclusions

This paper presents a novel accurate frequency domain panoramic imaging algorithm for ArcSAR. The proposed algorithm takes advantage of the shift-invariant property of the angular signal, and the main steps are all performed in the angular frequency domain, so that the 360° scanning raw data can be focused at one time without dividing the data into narrow angle data. In the proposed algorithm, the matched filter in 2-D frequency domain is accurately deduced for the reference range, and the range variant correction function in the range and angular frequency domain is also accurately deduced. This ensures that this algorithm is not only efficient, but also very accurate, and anhigh-resolution wide-swath panoramic image can be obtained without the segmenting strategy.

The advantages of the proposed algorithm over the other algorithms are validated by both simulation and real data. The 360° field of view imaging capability is validated by simulation, but only partially validated by the real data. The system that is used in the field test is still an experimental system, and the rotating angle is about ±80°. In the following work, we will improve the system to support 360° field of view imaging. The results, both simulation and real data, show that our algorithm brings the quality of image close to the time domain BP algorithm at a processing efficiency that is approximately two orders of magnitude better, and it has better image quality than the existing frequency domain Lee’s algorithm at a comparable processing speed.

## Figures and Tables

**Table 1:** Experimental parameters.

Parameter	Value
*r* (m)	1
$\theta_{\mathrm{bw}}$ (rad)	π/3
$f_{c}$ (GHz)	17
$B_{r}$ (GHz)	1

**Table 2:** Image quality test.

Target	Angular Imaging Quality	BP	Our Method	Lee’s Method
Near range target	IRW (°)	0.4506	0.4656	- -
	PSLR (dB)	−12.3226	−12.8166	- -
	ISLR (dB)	−9.1585	−9.5276	- -
Central range target	IRW (°)	0.4506	0.4656	0.5257
	PSLR (dB)	−12.4066	−12.8807	−11.3525
	ISLR (dB)	−9.2485	−9.6129	−7.1747
Far range target	IRW (°)	0.4506	0.4656	0.5257
	PSLR (dB)	−12.3956	−12.8705	−11.0764
	ISLR (dB)	−9.2374	−9.5558	−6.9512

**Table 3:** Real data parameters.

Parameter	Value
*r* (m)	1
$\theta_{\mathrm{bw}}$ (rad)	π/3
$f_{c}$ (GHz)	17
$B_{r}$ (GHz)	0.3
$f_{s}$ (MHz)	60
$T_{p}$ (μs)	60
